# Using Species Groups to Approach the Large and Taxonomically Unresolved Freshwater Fish Family Nemacheilidae (Teleostei: Cypriniformes)

**DOI:** 10.3390/biology11020175

**Published:** 2022-01-22

**Authors:** Tomáš Dvořák, Vendula Šlechtová, Jörg Bohlen

**Affiliations:** 1 Laboratory of Fish Genetics, Institute of Animal Physiology and Genetics, Academy of Sciences, 27721 Liběchov, Czech Republic; v.slechtova@iapg.cas.cz (V.Š.); bohlen@iapg.cas.cz (J.B.); 2 Department of Zoology, Faculty of Science, Charles University, 12800 Prague, Czech Republic

**Keywords:** biodiversity, evolutionary history, systematics, molecular marker, freshwater fish, pigmentation pattern

## Abstract

**Simple Summary:**

Since large animal families with unresolved taxonomy are difficult to study, it is better to focus on smaller units. In the present study, we test if we can define a restricted group of species within a large and unsorted fish family (Nemacheilidae). In the beginning, we selected 17 candidate species that share a rare and specific pigmentation pattern, and 29 comparative species with a different pattern. We studied their relationships and the ages of pedigree branches using molecular genetic markers. It turned out that the candidate and comparative species are intermixed in two main groups, meaning that the specific pigmentation pattern is not diagnostic. However, the genetic lineages themselves are smaller units within the large family Nemacheilidae and can be used for further studies. For example, one group revealed its evolutionary history: it expanded 12 million years ago from India into Myanmar and later further to Thailand, Laos, and China. They also revealed more than 20% of undescribed species, a very high rate in vertebrates. Our results show that species groups can be a helpful tool to approach large and unsorted animal units. This finding offers species groups as a new tool for future studies exploring the diversity of life on earth.

**Abstract:**

Large animal families with unresolved taxonomy are notoriously difficult to handle with respect to their biodiversity, systematics, and evolutionary history. We approach a large and taxonomically unresolved family of freshwater fishes (Nemacheilidae, >600 species) by proposing, on the basis of morphologic data, a species group within the family and study its phylogeny with conclusions regarding its diversity, taxonomy, and biogeographic history. Phylogenetic analyses of two mitochondrial and three nuclear genes of 139 specimens, representing about 46 species (17 candidate species from the proposed species-group, plus 29 comparative species), revealed that the proposed species group does not form a distinct monophyletic lineage, but that the candidate and comparative species mixed in three different lineages. However, the results revealed more than 20% of undescribed species within the ingroup and showed that species do not cluster according to the presently recognised genera. At least one of the genetic clades shows signs of an eastward range expansion during the second half of Miocene from north India via Myanmar into Laos, western China, and western Thailand. We conclude that the approach of picking monophyletic lineages to study biodiversity, systematics, and evolutionary history helps to open the door to large animal families.

## 1. Introduction

The identification of phylogenetic relationships and groups of closely related taxa remains a major task in the study of biodiversity and evolutionary history. Predominantly, morphologic similarity was taken as a measure for the degree of relatedness between taxa, and species groups (often ranked as genus) were postulated for taxa that shared an otherwise absent morphological character state (synapomorphy) [1]. Indeed, characters of external morphology still provide valuable information to identify species groups and are unreplaceable for fieldwork. However, molecular genetic markers are nowadays considered as standard tools for species delimitation and revealing phylogenetic relationships among taxa (e.g., [2,3,4,5,6,7,8]). In optimal cases, the phylogenetic topologies revealed by morphological and genetic markers are congruent [9,10,11], leading to strong reciprocal support. However, many examples have been published where both datasets result in conflicting topologies (e.g., [12,13,14,15,16,17]), generating a need to explain the mechanism that would be responsible for the conflict.

Here, we want to highlight the importance of species groups in cases of large and taxonomically unresolved animal groups. As they provide the first hypothesis about certain distinct and monophyletic clusters within an unclarified mass, morphologically based species groups can act as stepping stones towards taxonomical understanding. One important advantage of these groups is their manageable size in comparison to a large unresolved group, as seen in *Drosophila* flies [18,19,20].

An example of a sizeable and taxonomically complex animal group is the freshwater fish family Nemacheilidae (Cobitoidea: Teleostei). It comprises small (usually <12 cm total length) benthic species that occur mainly in streams and rivers, but can also inhabit caves, swamps, or lakes. Nemacheilidae are distributed across the majority of Europe and Asia and are quite common, being found in nearly every river basin [21,22,23]. This omnipresence makes them one of the most characteristic elements of the Eurasian freshwater fauna and, as recent studies have shown, highly suitable model organisms for the study of evolutionary history and biogeography (e.g., [17,24,25]). With more than 700 species in roughly 50 genera it is a rich and diverse family, but with the number and outline of genera far from being settled [23,26,27,28,29].

Within the last decade, several attempts have been made to identify distinct monophyletic species groups within Nemacheilidae in South and Southeast Asia. For example, studies of morphologic similarities led to the descriptions of the genera *Mustura* and *Rhyacoschistura* [28,29], *Pteronemacheilus* [30], and *Paracanthocobitis* [31]. Genetic studies have been carried out addressing the *Paracanthocobitis zonalternans* group [24], the *Schistura robertsi* group [25], and the genus *Nemacheilus* [17]. In the last three examples especially, the genetic data not only helped to define the outline of a monophyletic species group but also to further investigate in detail their biogeographic and evolutionary history, proving the usefulness of species groups as model taxa.

In particular, the largest genus, *Schistura*, has long been shown by morphologic data [26,27,32], and more recently by genetic data [33,34,35,36,37], to be a polyphyletic artificial assemblage. However, due to its large size (~250 species) and wide distribution throughout South and Southeast Asia, no comprehensive phylogenetic overview has been produced up to now, leaving severe taxonomic uncertainties across *Schistura* and most other genera of Nemacheilidae in the region.

**Figure 1:**
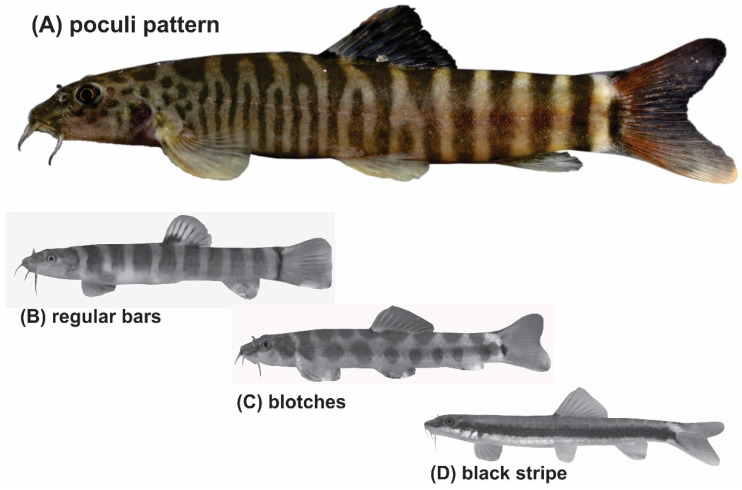
(**A**) The poculi pigmentation pattern consists of densely set narrow bars on the body sides between head and dorsal-fin origin and less densely set broader bars between the dorsal-fin base and caudal-fin base. It is found in only a few species of *Schistura, Physoschistura,* and *Mustura* (example species is *Schistura mahnerti*). The vast majority of species in these genera show other pigmentation types like (**B**) bars of similar width along the whole body (*Schistra robertsi*), (**C**) blotches (*Schistura* corica), or occasionally (**D**) longitudinal stripes (*Schistura pawensis*).

Taking morphologic characters as a guide, more species groups might be seen within *Schistura* and other genera in South and Southeast Asia. Indeed, we postulate the presence of a ‘*Schistura poculi* group’; a newly identified group collecting all species sharing the same striking pigmentation pattern. As in most species of *Schistura,* the suspected members of the *Schistura poculi* group bear regular bars on the body from the back across most of the side, but in these certain species, the bars between the head and the level of dorsal-fin are numerous, very narrow, and densely set, while the bars behind the dorsal fin are few, but sparsely set (Figure 1). We will further refer to this specific pigmentation pattern as the ‘poculi-pattern’. The poculi-pattern is generally rare among Nemacheilidae and present in only 20–25 species of *Schistura,* plus about 5 species from the genera *Mustura* and *Physoschistura*. Nearly all of these species live in a coherent geographic area that stretches from northeast India through Myanmar to southwest China and the western parts of Thailand and Laos. The pigmentation pattern has recently been shown to be indicative of the three largest genetic clades in another nemacheilid genus, *Nemacheilus* [17], and the distinctive feature of black blotches on the lower lip to be indicative of the *Schistura robertsi* species group [25].

The similarity in pigmentation pattern and the geographic proximity of their occurrence prompted the hypothesis that the species with the poculi-pattern represent another monophyletic species group within Nemacheilidae. If so, such a group with its distribution in the northeast Indian and the western half of the Indochinese region would be an appreciated model to study the biogeographic history of this area that played a key role in the colonisation of India. In the present study, we test this hypothesis using sequence data of two mitochondrial and three nuclear genes.

## 2. Materials and Methods

### 2.1. Sampling

**Figure 2:**
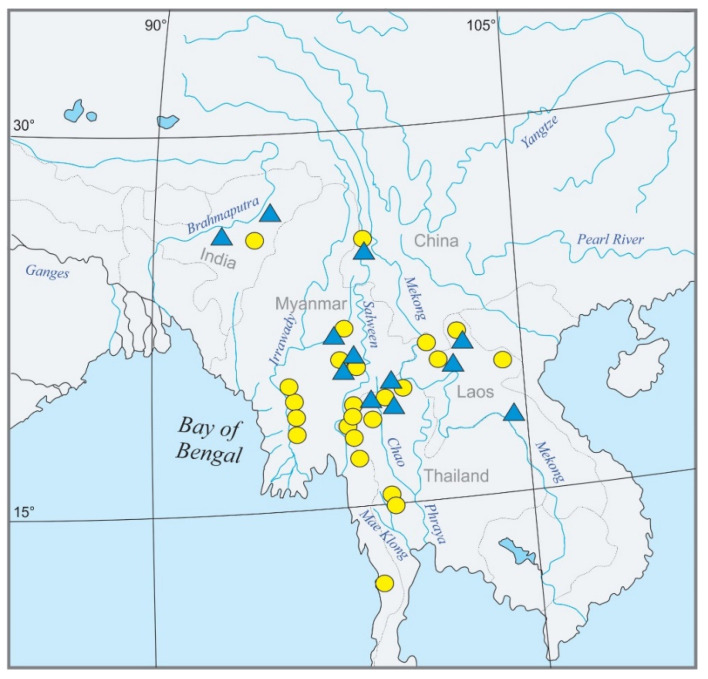
Geographic map of Southeast Asia showing the sampling sites for the present study. Candidate species are marked by yellow circles, ingroup species with different pigmentation patterns by blue triangles. A mark may represent several localities.

The fish species originated from northeast India, Myanmar, southwest China, Laos, and western Thailand. An overview of the geography of the region and the sampling points for the present study is given in Figure 2. The molecular analysis is based on 139 specimens covering about 46 species from the genera *Mustura, Petruichthys, Physoschistura, Pteronemacheilus,* and *Schistura* (Table S1). Among the selected taxa were 17 candidate species for the proposed *Schistura poculi* group, meaning species that bear the poculi-pattern of pigmentation. These candidate species are presently assigned to three different genera (*Mustura, Physoschistura,* and *Schistura*). In a first pilot analysis (data not shown), the candidate species were analysed together with a larger number of Nemacheilidae species with different pigmentation patterns and, according to the results, the 14 species found to be closely related with the candidate species were selected and joined with them to form the ingroup in our analyses. We then selected 15 distantly related species of Nemacheilidae and one species of Cobitidae as an outgroup to allow for calibration of the dataset. It was found that all ingroup species with different pigmentation occur in the same geographic area as the candidate species.

### 2.2. Laboratory Procedures

DNA was extracted from fin or muscle tissue either by Dneasy Blood & Tissue kit (QIAGEN N.V., Venlo, Netherlands) or by the phenol-chloroform method [38]. As an outgroup, we used published sequences of various Nemacheilidae and *Cobitis taenia* from the closely related loach family Cobitidae [24,25,35].

For the present study, we used two mitochondrial (mtDNA) genes –cytochrome oxidase I (CO1) and cytochrome *b* (Cyt *b*) and three nuclear (nDNA) genes-interphotoreceptor retinoid-binding protein gene 2 (IRBP 2), recombination-activating gene 1 (RAG 1), and myosin heavy chain 6 (MYH6). The list of primers used for amplification and/or sequencing is provided in Table 1.

PCRs were performed in 25 μL reaction volumes containing 10 mM Tris–HCl, 50 mM (HN4) 2SO4, 0.1% Triton X-100, 1.2–1.8 mM MgCl2 (PCR Blue Buffer), 2 mM TMA oxalate (PCR enhancer), 5 nmol of each nucleotide (PCR dNTP mix), 1.25 U of Taq DNA polymerase Unis (all chemicals by Top–Bio s.r.o., Vestec, Czech Republic), and 12.5 pmol of each primer.

The PCRs were carried out on a DNA Engine Peltier Thermal Cycler (BioRad Laboratory Inc., Hercules, CA, USA). For CO 1, Cyt *b* and RAG 1 we used a program with a touch-down (TD) profile of 1 min 30 s at 60–55 °C (1 °C/cycle) and 2 min at 72 °C, followed by 30 cycles with annealing temperature held at 54 °C. The amplification of IRBP 2 included 35 cycles with annealing temperature at 57 °C for primer combination 101F and 1162R and 59 °C for primer combination 109F and 1001R, 109F, and 1162R. The amplification of MYH 6 included 35 cycles with annealing temperature at 53–57 °C. Results of PCRs were checked on 0.8% agarose gel.

Most PCR products were processed by the professional sequencing service MACROGEN Europe BV (Amsterdam, Netherlands), with the remainder purified by QIAquick PCR purification kit (QIAGEN) and sequenced on an ABI Prism 3130 GA. For direct Sanger sequencing of PCR products, we used BigDye™ Terminator Cycle Sequencing Ready Reaction Kit 1.1 (Applied Biosystems, Bedfort, MA, USA). The preparation of the reaction mixture and the cycling profile followed the manufacturer’s instructions. Sequencing reactions were purified using DyeEx 2.0 Spin Kit (QIAGEN).

### 2.3. Phylogenetic Analyses

#### 2.3.1. Assembling and Alignment

Chromatograms were checked and assembled in the SeqMan II module of the Lasergene software package (DNASTAR Inc., Madison, WI, USA). Single-gene alignments were done in BioEdit [45] with the use of ClustalW [46] multiple alignment algorithms. The datasets were concatenated in PhyloSuite v1.2.2 [47]. The lengths of the final alignments were 682 bp for CO 1, 1134 bp for Cyt *b*, 952 bp for RAG 1, 881 bp for IRBP 2, and 782 bp for MYH 6. The concatenated dataset of mtDNA genes (Cyt *b* and CO 1) was 1816 bp long and the concatenated dataset of nDNA genes (RAG 1, IRBP 2, and MYH 6) was 2615 bp long. The mtDNA and nDNA datasets could not be concatenated due to strong discrepancies.

Ready alignments were imported to MEGA v. 7.0 [48] where the first Neighbour-Joining trees were prepared to check the datasets for potential sample mix-ups. In MEGA v. 7.0 the numbers of variable and parsimony-informative positions were obtained and the genetic differences between the lineages were counted. To allow easy comparison and comparability with other studies, we used the proportional genetic difference converted into percentages.

#### 2.3.2. Phylogenetic Trees

The best-fit substitution models and partitioning schemes were estimated using Partition Finder 2 [49] (implemented in PhyloSuite 1.2.2; [47]) based on the corrected Akaike Information Criterion (AICc).

The phylogenetic analyses were performed using the Bayesian inference (BI) and maximum likelihood (ML) (final original trees in supplementary file). The genes were analysed separately as well as in two concatenated datasets: one for the mitochondrial and one for the nuclear genes. For BI we used MrBayes 3.2 [50] via CIPRES Science Gateway [51]. The datasets were partitioned into genes and codon positions and the partitions parameter settings corresponding to the best-fit models were assigned. Model parameters were unlinked across the partitions. Each of the analyses was performed in two parallel runs of 10 million generations with 4 Metropolis Coupled Markov Chains Monte Carlo (MCMCMC) of default heating conditions. The sampling frequency was set to every 100 generations. The results were checked in Tracer v 1.7.1 [52] to determine the effective sampling size (ESS) of the parameters. Additionally, the stationarity of the log-likelihood scores was checked by plotting them against the generations. The relative burn-in of 25% was used and from the remaining 150,002 trees, 50% majority rule consensus trees were constructed.

The partitioned ML analysis was performed using IQ-TREE [53] implemented in PhyloSuite, using the estimated loci and codon position-specific models. The node support values were obtained with 1000 ultrafast bootstrap replicates (UFBoot) [54].

#### 2.3.3. Species Delimitation and Species Trees

For the species delimitation, we used Automatic Barcode Gap Discovery (ABGD) [55] and Assemble Species by Automatic Partitioning (ASAP) [56] (both distance-based methods) on the alignment of CO1 sequences (ABGD + ASAP) and a concatenated mtDNA dataset (ASAP). The ABGD species delimitation was performed via the ABGD web server with default settings and testing of all three substitution models for calculation of the distances (https://bioinfo.mnhn.fr/abi/public/abgd/abgdweb.html, accessed on 10 January 2022) and ASAP species delimitation was performed via the ASAP web server with testing of all three substitution models for calculation of the distances (https://bioinfo.mnhn.fr/abi/public/asap/asapweb.html, accessed on 10 January 2022). For tree-based methods for species delimitation, the General Mixed Yule Coalescent (GMYC) [57] and Bayesian Poisson Tree Process (bPTP) [58] approaches were applied. Due to the mode of inheritance, the mitochondrial genes can be functionally considered as one locus, therefore GMYC and bPTP approaches were applied on the ultrametric BEAST tree of concatenated mtDNA dataset of ingroup taxa. The GMYC species delimitation was performed via the GMYC web server (https://species.h-its.org/gmyc/, accessed on 10 January 2022) using a single threshold setting. The bPTP analysis was done on the bPTP web server (https://species.h-its.org/ptp/, accessed on 10 January 2022) with default settings.

For reconstruction of the species tree, we used a multi-species coalescent approach in StarBEAST2 [59], implemented in BEAST 2.6.0 [60]. In order not to oversplit the dataset, we used the rather conservative results of the ABGD analyses for the grouping of individuals into species with the exception that, due to their different distribution areas, we kept three genetic clades for *S. poculi* while the ABGD suggested joining them. For the final analyses, we used the species tree UCLN (uncorrelated lognormal clock) template and Yule model in priors. Due to the strong incongruence between the mtDNA and nDNA, the two sets of alignments had to be analysed separately. We ran the analyses for 500 million generations with a sampling frequency of 50,000 and checked the resulting log-files in Tracer v 1.7.1 [52] to see if the ESSs of all parameters were sufficient (>200) and the final species trees (as maximum clade credibility trees) with 20% burn-in were produced in TreeAnnotator 1.6.1 [61]. The topologies of the resulting mtDNA and nDNA based trees were compared in Dendroscope 3 [62].

### 2.4. Divergence Time Estimation

The ages of cladogenetic events were estimated from the concatenated mtDNA dataset as well as from the concatenated nDNA dataset in BEAST 2.6.0 [60] via CIPRES Science Gateway. For calibration, we used the same two calibration points as Bohlen et al. [24]: The first is based on the only known nemacheilid fossil record, *Triplophysa* *opinata* from Kyrgyzstan from the middle-upper Miocene (16.0 to 5.3 mya) [63,64]. The record sets the minimum age of the genus *Triplophysa* to 5.3 mya. For the second calibration point, we used the potential age of the Indian Nemacheilidae (45–24 mya). At the earliest, 45 mya the Indian subcontinent had made surface contact with the Asian mainland, allowing freshwater fishes to colonise it. At 24 mya, the river connection between India and mainland Asia was broken by the uplift of the Himalayan Mountains and the Tibetan Plateau; since then, the Indian freshwater fauna has been isolated from the fauna on the Asian mainland [65].

BEAST was lacking a standard tree prior for datasets containing interspecific and intraspecific taxa and only a few recommendations for a preference of the Birth-Death model exist [66]. Therefore, our standard approach was to compare the performance of different tree priors with the same settings and choose the one with the best performance. In the present case, we compared the analyses using Yule and Birth-Death tree priors, both with various settings. In all cases the resulting topologies and estimated ages were the same or very similar, however, the analyses with the Yule tree prior provided better ESS and posterior probabilities so the Yule model was used in the final analyses.

For the final analyses, the partitions were unlinked and assigned the estimated evolutionary models. As priors, the Yule process of speciation and relaxed lognormal molecular clock were selected for the analyses (final original trees in supplementary file). The origin of the genus *Triplophysa* was based on the mentioned fossil record and set to 5.3–16 mya (based on the estimated age of *T. opinata*) with the use of fossil prior. The calibration point of the “Indian lineage” was set to uniform distribution 24–45 mya. The MCMC analyses were set to 5 × 10^6^ generations with a sampling of every 5000 generations. The outputs were checked in Tracer 1.7.1 [52] to see if the effective sampling sizes (ESS) for all parameters were sufficient (>200). The maximum clade credibility (MCC) tree was built in TreeAnnotator 2.6.0 [61] after discarding the first 10% of trees as burn-in. The final trees were visualised in FigTree 1.4.4 [67].

## 3. Results

### 3.1. Phylogeny

The phylogeny of the *Schistura poculi* group was reconstructed using 136 sequences of Cyt *b* (1133 bp), 136 sequences of CO 1 (682 bp), 137 sequences of RAG 1 (952 bp), 132 sequences of IRBP 2 (881 bp), and 137 sequences of MYH 6 (782 bp). The concatenated mtDNA and nDNA datasets were composed of 272 sequences (together 1815 bp) and 406 sequences (2615 bp), respectively. An overview of the data characteristics including lengths, percentages of variable and parsimony-informative positions as well as substitution models is given in Table 2.

The pairwise lineage genetic differences (Table S2) within the *Schistura poculi* group range in the concatenated mitochondrial alignment (Cyt *b* and CO 1) from 2.18% to 8.07%. In the combined dataset of the nuclear genes RAG 1, MYH 6, and IRBP 2, the genetic differences ranged from 0.09% to 1.14%.

**Figure 3:**
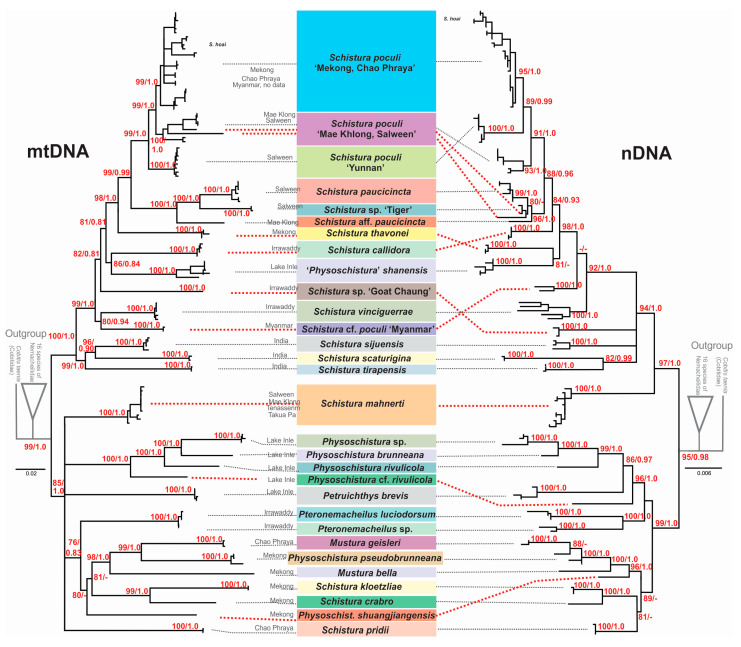
Maximum likelihood trees of the loach species around *Schistura poculi* based on two mitochondrial (**left**) and three nuclear (**right**) markers. Support values at the nodes are ML/BI values. Nodes with support <75 and intraspecific support values are not shown. Cases of conflict are indicated by dotted red lines.

Since single-gene analyses (data not shown) resulted in almost congruent topologies for the two mitochondrial genes Cyt *b* and CO 1, and no significant incongruencies were detected among the three nuclear genes RAG 1, IRBP 2, and MYH 6, we prepared a concatenated mitochondrial tree and a concatenated nuclear tree (Figure 3). The phylogenies reconstructed using ML and BI approaches were entirely identical. However, between the mitochondrial and nuclear trees, several significant discrepancies were found that hampered the concatenation of all the data and must be taken into account in the following summarisation of results and the discussion.

**Figure 4:**
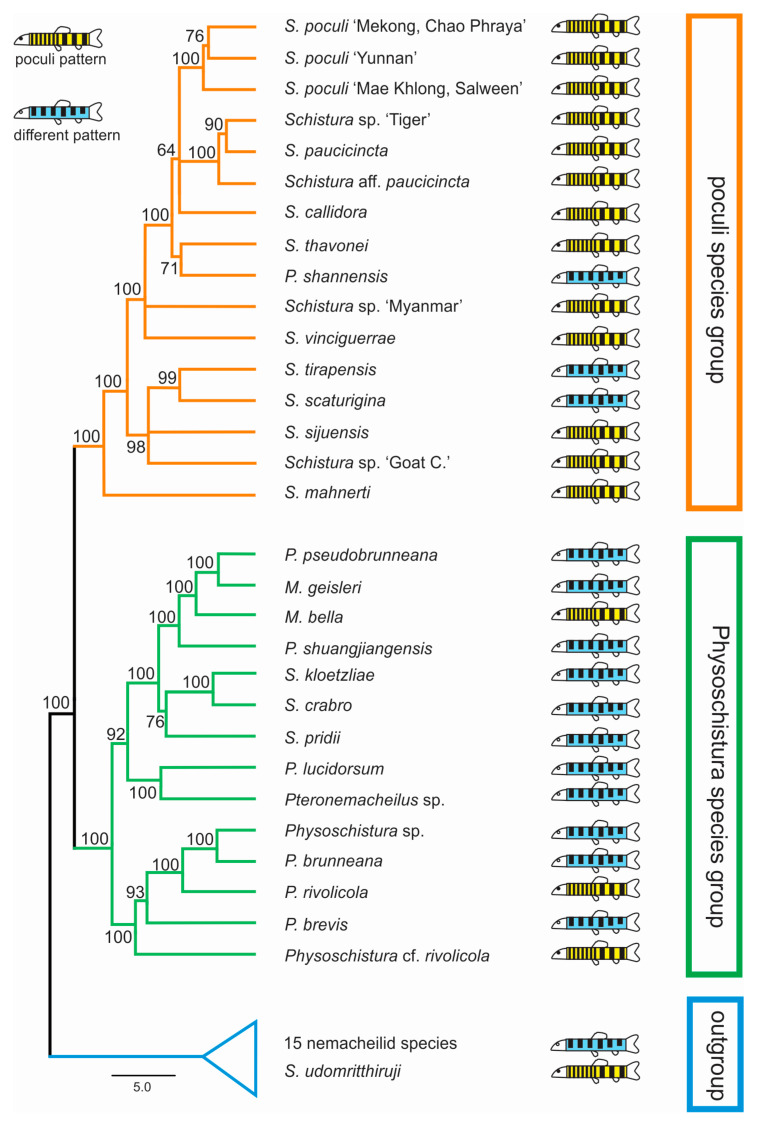
Distribution of candidate species within the phylogeny (ultrametric tree of the concatenated nuclear dataset generated in BEAST). The resulting phylogeny shows two major clades, here named ‘poculi species group’ and ‘Physoschistura species group’, plus the outgroup. Candidate species (those with the poculi pattern, yellow symbols) are found in both species groups and the outgroup and are intermixed with species with different pigmentation patterns (blue symbols).

In all analyses, three groups were identified in both datasets: the first included 12 of the 17 candidate species, plus 3 species with different pigmentation; the second had 4 candidate species, plus 11 species with different pigmentation; and the third group contained 1 candidate species, plus 15 nemacheilid species that were included for callibration purposes only. The first two groups have sister-relation and together represent the ingroup of our study, while the third group is considered the outgroup. Within the ingroup, species with and without poculi patterns were mixed (Figure 4).

### 3.2. Time Estimation

**Figure 5:**
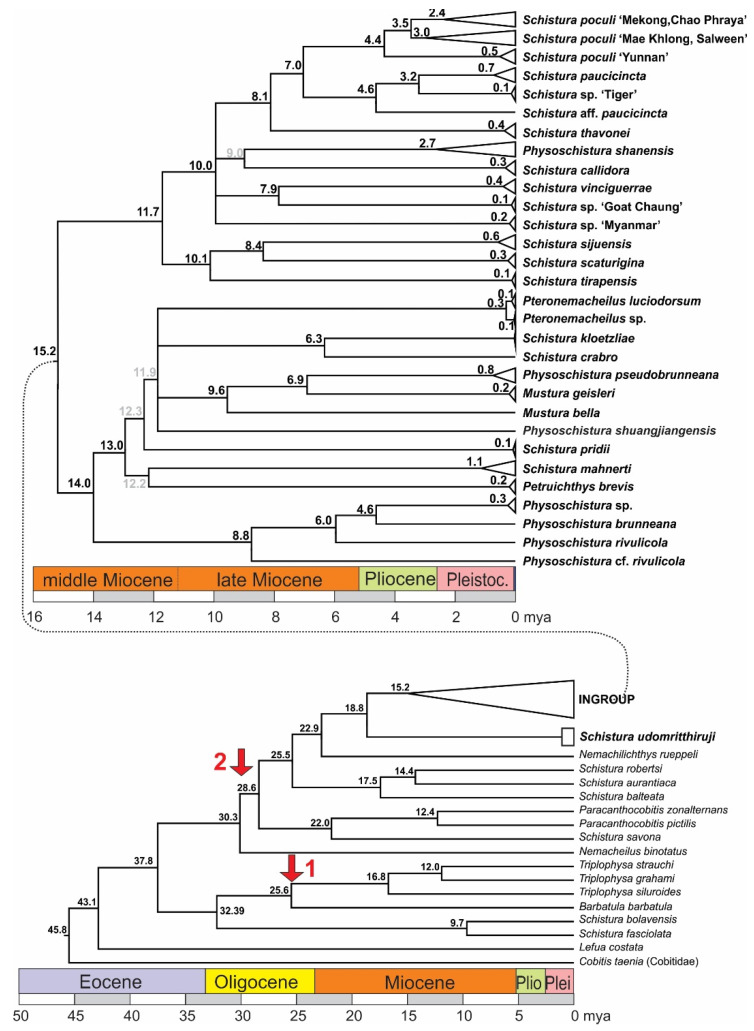
Time tree of the mitochondrial dataset and calculated ages of the genealogic events. Nodes with support <50 are collapsed; at nodes with statistical support between 50 and 75 the colour of the age numbers is grey. Red arrows mark the calibration points: arrow 1 marks the age of the genus *Triplophysa*, to which the only fossil species of Nemacheilidae belongs, and arrow 2 the origin of the Indian radiation.

The time tree of analysed samples dated the origin of Nemacheilidae to about 45 mya and the separation of the ingroup from the outgroup to about 18.8 mya (Figure 5). Within the ingroup, an important split into two major clades took place about 15.2 mya. In one group, a lineage that led to four species of *Physoschistura* separated around 14 mya while the remaining species in this group developed in the time period of 13 to 6 mya. In the other group, the radiation started around 12 mya and most lineages had formed around 8 mya, but some younger genealogic events show that radiation is still in process.

### 3.3. Species Delimitations and Species Trees

The ABGD species delimitation approach based on the COI dataset divided the individuals into 27, 29, and 31 groups (13 in the Schistura poculi group vs. 14–18 in the Physoschistura group), where the differences were only within the clades formed by *S. mahnerti* and *Pteronemacheilus*. The ABGD provided 3 different possibilities of grouping based on maximal distance prior (for maximal distance *p* = 0.001000 31 groups, for maximal distance *p* = 0.001668 and *p* = 0.002783 29 groups, for maximal distance *p* = 0.004642–*p* = 0.035938 27 groups). The ASAP approach provided very similar results as ABGD, the best partition divided the COI dataset into 27 groups (13 in the Schistura poculi group vs. 14 in the Physoschistura group) and the concatenated mtDNA dataset into 25 groups (11 in the Schistura poculi group vs. 14 in the Physoschistura group). The GMYC and bPTP approaches both divided the individuals into 45 groups (28 in the Schistura poculi group and 17 in the Physoschistura group). In the bPTP approach, no differences were present between the results of ML and BI solutions.

**Figure 6:**
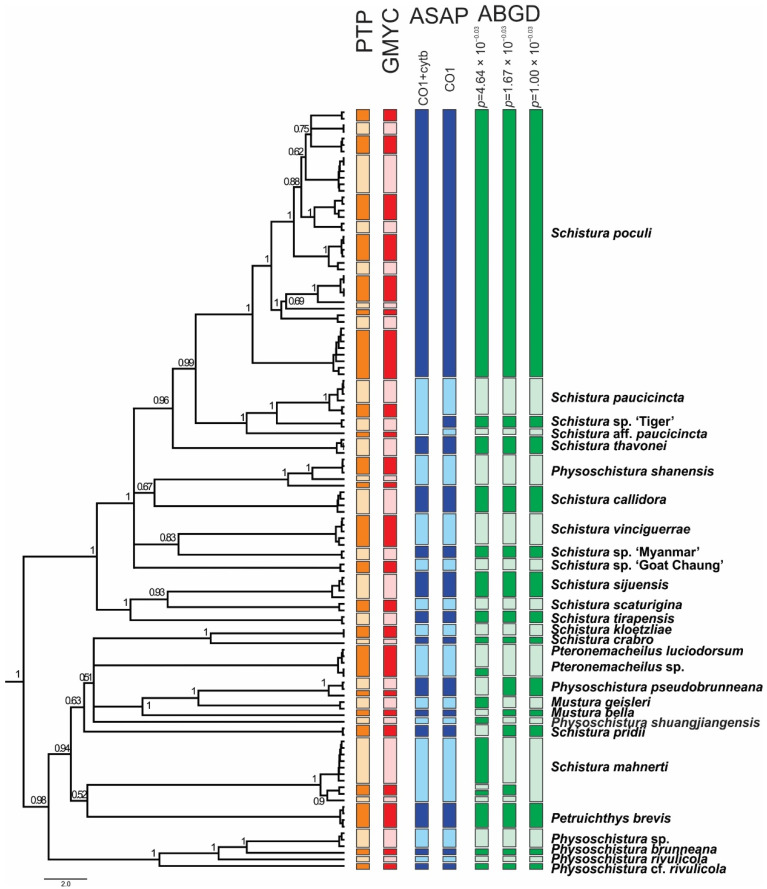
Ultrametric BEAST tree of the concatenated mitochondrial dataset, with a graphical explanation of results of various species delimitation analyses. The two different results of the ASAP approach correspond with using only the COI dataset vs. concatenated mt DNA dataset (COI + Cyt *b*). The three different results of the ABGD approach correspond to different possibilities of grouping based on maximal distance prior. Nodes with support <50 are collapsed.

For the species delimitations, the tree-based methods (GMYC and bPTP) suggested higher variability within the group than the distance-based methods (ABGD and ASAP). The difference is more evident in the poculi Schistura poculi group, which contains a higher intraspecific variability than the Physoschistura group, with mainly interspecific variability. The results of applied species delimitation methods are visualised in Figure 6.

**Figure 7:**
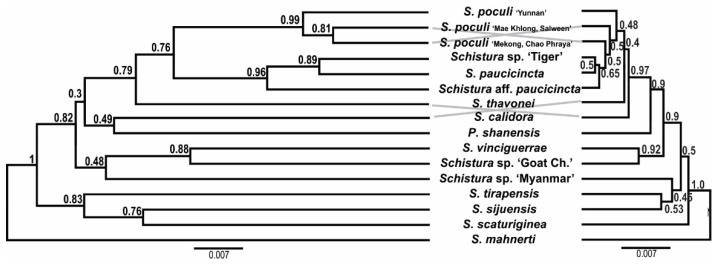
Tanglegram of the species trees based on mtDNA (**left**) and nDNA (**right**); both trees are proportional. The poor statistical support and short branch length in the nDNA tree are most likely the result of ancestral polymorphism and incomplete lineage sorting and/or horizontal gene flow between lineages. *Schistura mahnerti* was used to root the trees.

The tanglegram of the mtDNA and nDNA species trees is shown in Figure 7. The species tree topologies are very similar to the topologies seen in the phylogenetic trees of these datasets. However, the comparison of both species trees in the same scale visualises the lower resolution of the nDNA dataset by the shorter branch lengths and the lower support values.

## 4. Discussion

### 4.1. Is there a Monophyletic Schistura Poculi Species Group with a Unique Pigmentation Pattern?

Our results show that the candidate species, i.e., the 17 analysed species that bear the poculi-pattern, do not form a single monophyletic group. Instead, they are intermixed with species with different pigmentation patterns in three different groups (Figure 4). This result means that the poculi-pattern itself is not diagnostic or exclusive for any species group. We cannot conclude from our data if the poculi pattern evolved several times independently in the three clades or if it was secondarily lost in the species with different patterns in the same clades, but it is clear that it is not a diagnostic character for any of the genetic lineages in the results.

However, our results do identify two distinct and monophyletic species groups within the analysed part of Nemacheilidae, even if there is presently no simple morphologic character that would be able to diagnose them. The first of these groups collect twelve species with the poculi-pattern, plus three species with other patterns. Therefore, the clade is dominated by species with the poculi-pattern and includes *S. poculi*, and so will now be referred to as the ‘poculi species group’. The second group contains four species with the poculi-pattern and eleven species with different patterns. The genus with the most species within this group (six species) is *Physoschistura* including its type species, *P. brunneana*. Since *Physoschistura* is also the oldest available name in this group, we will refer to this clade as the ‘Physoschistura species group’. We found that one candidate species, *Schistura udomritthiruji*, even belongs outside of the two species groups.

Apart from the poculi-pattern that was a priori assumed as a synapomorphy of the predicted *Schistura poculi*-group, we did not find any synapomorphies in the poculi- and Physoschistura species groups. The morphologic differences between the analysed species in this study ranged from small differences in pigmentation pattern, e.g., between *Schistura poculi* and *Schistura hoai,* to very large: *Petruichthys brevis* is a pelagic lake inhabitant, while all others are benthic stream inhabitants. Some species have a complete and some an incomplete lateral line; they have different fin and mouth shapes, different fin ray numbers, and a sexual dimorphism is present or absent, but no character state of these morphological characters is diagnostic for one of the species groups (data not shown). Contrastingly, the fish genus *Tropheus* in Lake Tangayika have nearly identical body shapes and differ only in pigmentation [68,69]. *Schistura* and related genera do have characteristics that differentiate between species, but at present it is unclear which are phylogenetically informative in a higher taxonomic frame. A lot of taxonomic work has been dedicated by various specialists to sort the variety of Nemacheilidae into genera, but already our analysis of just a small part of Nemacheilidae shows that the traditionally used morphologic characters do not define the bigger genera well. This is why we investigated if the pigmentation pattern, a formerly untested character, would yield phylogenetic information.

Identifying and establishing two formerly unknown species groups increases our understanding and brings some order into the still unordered family Nemacheilidae. Since the monophyly of these groups has now been demonstrated, they immediately become available as models for further studies, e.g., on biogeography and evolution. We will now provide further details of the evolutionary history of the poculi species group.

In contrast to our expectations that nemacheilid loaches with the poculi-pattern represent a monophyletic group, we found the pattern to be present in at least two clades and that the diversity of the fishes in focus is, in fact, higher than formerly believed. In a similar way, recent genetic studies on the phylogeny of species groups in Nemacheilidae in Southeast Asia found more and different grouping patterns than expected. The species *Paracanthocobitis zonalternans* from eastern Myanmar and western Thailand were supposed either to represent a single species [27] or three species [70], however, a molecular data-based phylogeny showed it to be composed of six genetic clades, two of them sympatric, and the others allopatric [24]. A phylogenetic study on the *Schistura robertsi* species complex revealed at least ten species in the group instead of the four originally anticipated ones [25]. A phylogenetic investigation of the genus *Nemacheilus* revealed that at least five species do not belong to *Nemacheilus,* but to three different clades within Nemacheilidae [17]. The similarity of these outcomes demonstrates that the phylogenetic investigation of species groups can bring a valuable increase in the understanding of taxonomy.

### 4.2. Dating of Events and Biogeographic Signal in the New Poculi Species Group

**Figure 8:**
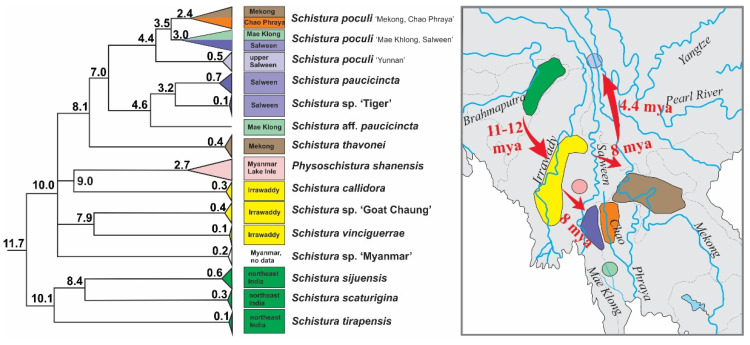
Part of time tree and geographic distribution of species in the poculi species group. The three species from northeast India were the first to become isolated, followed by the species in the Irrawaddy basin and Lake Inle in Myanmar. The radiation into the Mekong, Chao Phraya, Salween, and Mae Khlong then occured, indicating an eastward range extension of the group during the middle and late Miocene.

According to our results, the origin of the poculi species group (corresponding to its divergence from the Physoschistura species group) dates to about 15 mya and although we did not carry out a full biogeographic analysis, a clear biogeographic trend is visible (Figure 8): the oldest split within the group was dated to about 11–12 mya and separates the three species from north India (*S. tirapensis, S. sijuensis,* and *S. scaturigina*) from the remaining species, which all originated from localities east from India. The next younger phylogenetic events about 10 mya led to four lineages; three lineages collect all species from Myanmar (*Schistura* sp. ‘Myanmar’, *Schistura* sp. ‘Goat Chaung’, *S. vinciguerrae*, *S. callidora*, and *P. shanensis*), while the fourth lineage underwent another round of radiation at ~ 8 mya and afterward led to all species from Laos, China, and Thailand (*S. thavonei, S.* aff. *Paucicincta*, *Schistura* sp. ‘Tiger’, *S. paucicincta*, and three clades of *S. poculi*). We interpret this pattern as the result of an eastward expansion of the poculi species group during the second half of the Miocene from north India (Indian subregion according to Abbell et al. [71]) via Myanmar (Southeast Asian subregion, western Indochina province) into Laos (Southeast Asian subregion, Mekong province), western China (Southeast Asian subregion, Salween province), and western Thailand (Southeast Asian subregion, Salween province, and Thai Gulf province). Talking in biogeographic terms, the expansion crossed the border between the Indian and the Southeast Asian subregion, and within the later subregion led from the western Indochina province into the Mekong, Salween, and Thai Gulf provinces. Such massive range extension across the borders of provinces and even subregions is remarkable since it spans across the Arakan Mountains (including the Naga Hills, Chin Hills, Rakhine Yoma, and the Tenasserim Mountain Range including the Shan Plateau). These two north-south stretching mountain chains were uplifted by the lateral deformation of the Asian plate during the collision with the Indian plate [72] and are well-known biogeographic barriers for freshwater fauna (as found in sponges and Polychaeta [73], bivalve mussels [73,74] and fish [75,76,77,78]).

Two main mechanisms have been identified to enable range expansions in strict freshwater faunas: The first mechanism is by river capture, where a river erodes into the drainage of another river, causing headwaters of the invaded river system (and the fish within) to belong to the drainage system of the invading river. The second mechanism is by contact of the lower courses of rivers, which happens particularly during geologic periods with lowered sea levels. Both mechanisms have been shown to have shaped the present diversity of Nemacheilidae [24,25]. Since recent geologic studies deny a river contact between the north Indian Yarlung-Brahmaputra and the Burmese Chindwin-Irrawaddy during the Miocene [72,79,80,81], no potential colonisation pathway from north India to Myanmar is known. On the other hand, the separation of the Burmese species from the Indian species happened during the first dramatic drop of the global sea level during the Cenozoic (from about 50 m higher than presently, to about 80 m below today’s level) [82], which would prolong the rivers draining into the Bay of Bengal and potentially open a pathway for freshwater fishes. Such drops in the global sea level in general, and particularly the dramatic drop 10 mya, have been demonstrated to have supported faunistic range extensions and the colonisation of formerly inaccessible regions by Nemacheilidae [17,24]. We consider it a likely mechanism to have triggered the eastwards expansion of the poculi species group.

A very similar biogeographic pattern has also been found in freshwater mussels of the family Unionidae [74]: an Indian fauna west of the Arakan Mountains, a western Indochinese fauna between Arakan and Tenasserim Mountain ridges (drainages of Irrawaddy and Salween), and a Sundaland fauna (Mekong, Chao Phraya, and Mae Khlong). Interestingly, the diversification of Unionidae has occurred by far earlier (180–50 mya) than our study group (15–8 mya), showing that a biogeographic expansion over a vast region has occurred at least twice in different families of freshwater animals. Freshwater crabs of the family Potamidae have a much wider distribution than our study area, had their radiation during an earlier period (about 23–15 mya), and show a much larger number of genetic lineages [83], but in at least some points their biogeographic pattern resembles our results: a major split runs through Central Myanmar, separating an eastern from a western subfamily, resembling the separation of the Indian species from the more eastward ones. Within the eastern subfamily of Potamidae, a southwest Chinese lineage can be observed, and a clade with distribution in north Thailand, northeast Myanmar, west Laos, and southwest China resembles the distribution area of two of our clades.

### 4.3. Conflicts between mtDNA and nDNA Markers

In all analyses, certain cases of discrepancy appear between the mitochondrial and the nuclear dataset. Such incongruencies between the maternally versus biparentally inherited genes need specific attention as they might indicate important events in the evolutionary history of the studied animal, particularly hybridisation events [84,85]. In the phylogenetic trees (Figure 2) we have indicated five of these cases: Two specimens from the Thai Salween River basin that are in the mitochondrial dataset part of *S. poculi* ‘Mae Khlong, Salween’ have in the nuclear dataset a closer affiliation to *Schistura.* sp. ‘Tiger’, also from the Thai Salween River basin. Such conflict might be the result of incomplete lineage sorting in comparably young radiation or of horizontal gene flow (hybridisation) between *S. poculi* ‘Mae Khlong, Salween’ and *Schistura* sp ‘Tiger’ [86]. Our data cannot differentiate between the two mechanisms, but it would be an interesting topic for a future study since, up to now, no case of hybridisation in the genus *Schistura* or any other of the here analysed genera has been recorded. Two more cases are the different positions of *Schistura* sp ‘Myanmar’ and *Schistura* sp ‘Goat Chaung’. *Schistura* sp ‘Myanmar’ is in mitochondrial dataset sister to *S. vinciguerrae* and nuclear dataset sister to the “tipgroup” containing *S. thavonei*, *S. callidora*, *P. shanensis*, *S. paucicincta,* and *S. poculi*. *Schistura* sp ‘Goat Chaung’ is in mitochondrial dataset sister to the “tipgroup” mentioned above, but in the nuclear dataset, it is sister to the three Indian species (*S. sijuensis*, *S. scaturigina,* and *S. tirapensis*). Another case refers to the 12 my old species *S. mahnerti*. In the mitochondrial dataset, it is a member of the Physoschistura species group, but in the nuclear dataset, it belongs to the poculi species group. More cases are indicated in Figure 3. It appears as if the whole group in the nuclear dataset contains numerous cases of incomplete lineage sorting and horizontal gene flow due to the very complex palaeogeographic history of the region. However, these cases of conflict hampered the concatenation of mitochondrial and nuclear datasets for two reasons: First, it would blur potentially important hybridisation events, and second, the concatenation of such incongruent gene trees would result in significantly lower statistical support and artefacts in the topology [87].

### 4.4. Taxonomic Implications

The primary species delimitation followed the results of the ABGD analysis based on the CO1 dataset. The results of the ABGD analyses were used to assign samples to species for the species trees calculation in StarBEAST. For the poculi species group, we further constructed species trees, one for the mitochondrial and one for the nuclear dataset (Figure 6). A comparison of the mitochondrial and nuclear species trees shows a much lower resolution (shorter branch lengths, on average much lower support values) in the nuclear species tree than in the mitochondrial species tree. This situation is caused by the different modes of inheritance of the genes as well as by a comparatively young age of the analysed fish group. Potentially, cases of incomplete lineage sorting or hybridisation may have weakened the resolution in the nuclear tree [87].

The species delimitation analyses identified at least seven unnamed species in the ingroup material (*Schistura* sp ‘Tiger’, *Schistura* aff. *paucicincta*, *Schistura* sp. ‘Myanmar’, *Schistura*. sp. ‘Goat Chaung’, *Pteronemacheilus* sp., *Physoschistura* sp., and *Physoschistura* cf. *rivulicola*), which are supported by all (or most) species delimitation approaches (Figure 8). Finding already more than 20% of species unnamed is a high rate for a study on vertebrates, even in freshwater fishes. For comparison, Kottelat [88] in an overview about the Xe Kong drainage in Laos reported 11% of all fish species as new to science, with 14% (2 out of 14 species) of new species in the genus *Schistura*. Most likely, the diversity within our analysed material is even higher. The upper limit of potential diversity is probably marked by the tree-based methods of species delimitation (GMYC + bPTP) which identified 15 unnamed taxa (45%). However, tree-based methods of species delimitation are known to be less conservative than distance-based methods [57,58] and sometimes show a tendency for overspliting [89,90].

Examples of fishes with an unclear taxonomic state include one of the two specimens with conflicting position (see above under point 4.4), which has a morphologic appearance that is remarkably different from any other analysed species (the second specimen was not examined due to its juvenile stage) and could be the result of a past hybridisation event. Further, there is evidence that the more widely distributed species (particularly *S. poculi* ‘Mekong, Chao Phraya’, but potentially also *S. mahnerti* and/or *S. vinciguerrae*) reveal a hidden diversity when being addressed taxonomically. For example, two of the four subclades within the clade *S. poculi* ‘Mekong, Chao Phraya’ are formed by the specimens of *S. hoai* (Mekong basin) and *S. poculi* from the type locality (Chao Phraya basin). Moreover, the three clades of *S. poculi* might turn out to be different species if addressed taxonomically.

In addition to this high species diversity, our results show the earlier mentioned vagueness in the delimitation of genera. *Physoschistura shanensis* is not a member of *Physoschistura* (type species is *P. brunneana*) and the polyphyly of *Schistura* is evident even in this rather small number of species. However, the taxonomic vagueness was one of the motivations for this study, and the two species groups identified here bring a little order into this mess, even across the outline of genera.

## 5. Conclusions

This study reveals the presence of two species groups within the Nemacheilidae from western Southeast Asia.

At first glance there is no clear correlation between phylogeny and the pigmentation pattern, neither is there one with geographic distribution, taxonomic rank, or other indicators of biodiversity. However, closer inspection reveals clusters of species from geographically close areas, an eastward expansion in at least one species group, and a stochastic accumulation of the poculi pigmentation pattern in the poculi species group. The overall heterogeneity reflects the complexity of the evolutionary processes that shaped nemacheilid loaches in the studied area. This highly variable group of fishes underwent recent radiation in a geologically highly complex region. Cases of river capture and potentially incomplete lineage sorting or hybridisation events added to the mosaic character of the evolutionary history which hampered the clear evidence of single driving forces and can only be understood when looked at on the very local scale. This is not a mess, but evolution in action.

In such a complex situation, the application of species groups turned out to be the best-suited approach to bring some order. The two species groups identified in this study cross generic outlines and are not strictly bound to morphotypes; however, they represent the first step of ordering the complex diversity in the study area and are now available as models for biogeographic or taxonomic analysis.

## Figures and Tables

**Table 1:** List of primers used in the present study for amplification and/or sequencing.

Locus	Primer name	Primer Sequence (5′-3′)	Reference
CO1	Fish F1	TCA ACC AAC CAC AAA GAC ATT GGC AC	[39]
	Fish R1	*TAG ACT TCT GGG TGG CCA AAG AAT CA*	[39]
Cyt *b*	Glu-L.Ca14337–14359	GAA GAA CCA CCG TTG TTA TTC AA	[40]
	Thr-H.Ca15568–15548	*ACC TCC RAT CTY CGG ATT ACA*	[40]
	CB-L.Ca14975–14994	CAC GAR ACR GGR TCN AAY AA	[40]
	CB-H.Ca15057–15035	*TCT TTR TAT GAG AAR TAN GGG TG*	[40]
IRBP 2	101F	TCM TGG ACA AYT ACT GCT CAC C	[41]
	109F	AAC TAC TGC TCR CCA GAA AAR C	[41]
	1001R	*GGA AAT GCA TAG TTG TCT GCA A*	[41]
	1162R	*TGG TGG WCT TYA GGC ACT TGT*	[41]
RAG 1	RAG-1F	AGC TGT AGT CAG TAY CAC AAR ATG	[42]
	RAG-RV1	*TCC TGR AAG ATY TTG TAG AA*	[43]
MYH 6	myh6-F507	GGA GAA TCA RTC KGT GCT CAT CA	[44]
	myh6-R1322	*CTC ACC ACC ATC CAG TTG AAC AT*	[44]

**Table 2:** Overview of the datasets used for the molecular analyses of the *Schistura poculi* group: lengths of alignments, percentages of variable (VP), and parsimony-informative positions (PIP); best-fit substitution models for partitions (1st, 2nd, and 3rd codon positions of all genes) suggested by Partition Finder for the phylogenetic analyses performed by Maximum Likelihood (ML); and Bayesian Inference (BI) and time estimations by BEAST2. All parameters were estimated for the full datasets (three upper rows in every gene) as well as for the ingroup for StarBEAST2 analyses (lower row).

Locus	Length (bp)	Dataset	VP (%)	PIP (%)	Analyses	1st Codon Position	2nd Codon Position	3rd Codon Position
Cyt b	1133	Complete	50.0	44.9	ML	SYM + I + G	GTR + I + G	TIM + I + G
					BI	SYM + I + G	GTR + I + G	GTR + I + G
					Beast	GTR + I + G	GTR + I + G	TRN + I + G
		Ingroup	34.6	32.1	Beast	TRNEF + I + G	TRN + I	TRN + G
CO I	682	Complete	38.0	35.2	ML	GTR + I + G	HKY + I	TRN + I + G
					BI	GTR + I + G	HKY + I	GTR + I + G
					Beast	GTR + I + G	HKY + I	TRN + I + G
		Ingroup	27.0	23.8	Beast	TRN + I	HKY + I	TRN + G
RAG 1	952	Complete	35.2	27.7	ML	GTR + I + G	F81 + I + G	GTR + G
					BI	GTR + I + G	F81 + I + G	GTR + G
					Beast	GTR + I + G	HKY + I + G	GTR + G
		Ingroup	15.4	11.4	Beast	GTR + I	JC + I + G	GTR + G
IRBP 2	881	Complete	44.0	27.8	ML	SYM + G	GTR + G	GTR + G
					BI	SYM + G	GTR + G	GTR + G
					Beast	GTR + G	GTR + G	TRN + G
		Ingroup	11.5	8.51	Beast	TRN + I	TRN + I	TRN + G
MYH 6	782	Complete	30.2	21.0	ML	GTR + I + G	GTR + I	SYM + G
					BI	GTR + I + G	GTR + I	SYM + G
					Beast	TRN + I + G	GTR + I	SYM + G
		Ingroup	8.44	6.27	Beast	HKY + I	HKY	SYM + G

## Data Availability

All sequences have been deposited to GenBank. Accession numbers are given in detail in Supplementary Material.

## Supplementary Materials

The following are available online at https://www.mdpi.com/article/10.3390/biology11020175/s1, Table S1: Overview of analysed material, their geographic origin, voucher specimens, and GenBank accession numbers; Table S2: Percental genetic differences between lineages.
