# Supplementary material for: Using Species Groups to Approach the Large and Taxonomically Unresolved Freshwater Fish Family Nemacheilidae (Teleostei: Cypriniformes)
Source: Biology (Basel). 2022 Jan 22;11(2):175. doi: 10.3390/biology11020175 (PMC8869502; doi:10.3390/biology11020175)
Supplement: Supplementary file 1 [file biology-11-00175-s001.zip › biology-1505234-SI.pdf]

Supplementary Material

Table S1. Overview about analysed specimens, their collection numbers (CMK= Collection Maurice Kottelat, rest IAPG), geographic origin and Genbank accession numbers.

Species	Collection Number	Locality	Cyt b (m)	CO 1 (m)	RAG (n)	IRBP (n)	MYH 6 (n)
**S. poculi species group**							
'Physoschistura' shanensis	6773	Myanmar, Shan state, Inle lake basin, stream Thale-U; type locality	OL191220	OL191096	OL191469	OL345536	OL191340
	6774		OL191221	OL191097	OL191470	OL345537	OL191341
	6775		OL191222	OL191098	OL191471	OL345538	OL191342
	6649	Myanmar, Shan state, tributary of Inle lake	OL191213	OL191089	OL191462	OL345529	OL191333
	6615	Myanmar, Shan state, Inle lake basin	OL191212	OL191088	OL191461	OL345528	OL191332
Schistura callidora	3909	Myanmar, Shan state, Irrawaddy basin, Nam Paw stream at Hsipaw; Paratypes	OL191189	OL191059	OL191438	OL345507	OL191303
	3910		OL191190	OL191060	OL191439	OL345508	OL191304
	5826	Myanmar, Shan state, Irrawaddy basin, Nam Paw stream at Hsipaw; type locality	OL191206	OL191082	OL191455	OL345522	OL191326
	5827		OL191207	OL191083	OL191456	OL345523	OL191327
Schistura paucicincta	4490	Thailand, Tak prov., Salween basin, River Mea le Mau	OL191196	OL191069	OL191445	OL345514	OL191313
	4491		OL191197	OL191070	OL191446	OL345515	OL191314
	4856	Thailand, Tak prov., Salween basin, River Moei,	OL191199	OL191073	OL191448	OL345517	OL191317
	4873		OL191202	OL191076	OL191451	OL345518	OL191320
	4946		OL191203	OL191077	OL191452	OL345519	OL191321
	4947		OL191204	OL191078	OL191453	OL345520	OL191322
Schistura poculi	708	Thailand, Chiang Mai prov., Chao Phraya basin, small tributary of River Ping	OL191139	OL191003	OL191387	-	OL191250
	709		OL191140	OL191004	OL191388	OL345461	OL191251
	716	Thailand, Chiang Rai prov., Mekong basin, River Kok	OL191141	OL191005	OL191389	OL345462	OL191252
	718		OL191142	OL191006	OL191390	OL345463	OL191253
	719		OL191143	OL191007	OL191391	OL345464	OL191254
	720		OL191144	OL191008	OL191392	OL345465	OL191255
	721		OL191145	OL191009	OL191393	OL345466	OL191256
	1283		OL191160	OL191025	OL191409	OL345479	OL191272
	743	Thailand, Chiang Mai prov., Chao Phraya basin, Doi Inthanon; type locality	OL191146	OL191010	OL191394	OL345467	OL191257
	744		OL191147	OL191011	OL191395	OL345468	OL191258
	6843		OL191224	OL191100	OL191473	OL345540	OL191344
	749	Thailand, Chiang Mai prov., Chao Phraya basin, Mae Nam Taeng	OL191148	OL191012	OL191396	OL345469	OL191259
	750		OL191149	OL191013	OL191397	-	OL191260
	759		OL191150	OL191014	OL191398	OL345470	OL191261
	760		OL191151	OL191015	OL191399	-	OL191262
	1138	Thailand, Mae Hong Son prov., Salween basin, Mae Nam Rit	OL191157	OL191022	OL191406	OL345476	OL191269
	1284	Thailand, Mae Hong Son prov., Salween basin, River Pai	OL191161	OL191026	OL191410	OL345480	OL191273
	1286		OL191162	OL191027	OL191411	OL345481	OL191274

	2470	Thailand, Chiang Mai prov., Mekong, tributary of Mae Nam Fang	OL191166	OL191035	OL191416	OL345485	OL191281
	2471		OL191167	OL191036	OL191417	OL345486	OL191282
	2913	China, Yunnan prov., Salween basin, River Nan Ting	OL191171	OL191041	OL191421	OL345490	OL191286
	2914		OL191172	OL191042	OL191422	OL345491	OL191287
	3048		OL191181	OL191051	OL191431	OL345500	OL191296
	3049		OL191182	OL191052	OL191432	OL345501	OL191297
	3050		OL191183	OL191053	OL191433	OL345502	OL191298
	3012	Myanmar, Shan prov., Mekong basin, Pankawe Chaung,	OL191176	OL191046	OL191426	OL345495	OL191291
	3013		OL191177	OL191047	OL191427	OL345496	OL191292
	3045	China, Yunnan prov., Salween basin, small mountain stream	OL191178	OL191048	OL191428	OL345497	OL191293
	3046		OL191179	OL191049	OL191429	OL345498	OL191294
	3047		OL191180	OL191050	OL191430	OL345499	OL191295
	4202	Thailand, Tak prov., Mae Klong basin, Khwae Yai	OL191193	OL191065	OL191442	OL345511	OL191309
	4203		OL191194	OL191066	OL191443	OL345512	OL191310
	4857		OL191200	OL191074	OL191449	-	OL191318
	4859		OL191201	OL191075	OL191450	-	OL191319
	10894	Thailand, Tak prov., Salween basin, River Moei	OL191246	OL191134	OL191495	OL345562	OL191377
	11035		-	-	OL191497	OL345564	OL191379
Schistura hoai	CMK20881	Laos, Phongsali prov., Mekong basin, Nam Long	OL191227	OL191106	OL191476	OL345543	OL191350
	CMK20919	Laos, Phongsali prov., Mekong basin, Nam Ou	OL191230	OL191109	OL191479	OL345546	OL191353
	CMK20962_1	Laos, Phongsali prov., Mekong basin, Nam Noua	OL191228	OL191107	OL191477	OL345544	OL191351
	CMK20962_2		OL191229	OL191108	OL191478	OL345545	OL191352
	CMK25918_1	Laos, Houaphan prov., Mekong, Nam Khan, Nam Saat at Ban Nam Saat	OL191238	OL191126	OL191487	OL345554	OL191369
	CMK25918_2		OL191239	OL191127	OL191488	OL345555	OL191370
Schistura scaturigina	3925	India, West Bengal prov., Brahmaputra basin, River Rydak	OL191191	OL191061	OL191440	OL345509	OL191305
	3926		OL191192	OL191062	OL191441	OL345510	OL191306
Schistura sijuensis	3698	Ornamental fish trade	OL191184	OL191054	OL191434	OL345503	OL191299
	3699		OL191185	OL191055	OL191435	OL345504	OL191300
	3701		OL191186	OL191056	-	-	-
	11327		OL191248	OL191136	OL191499	OL345566	OL191381
Schistura thavonei	CMK26066	Laos, Louang Namtha prov., Mekong basin, Nam Ma Yen	OL191243	OL191131	OL191492	OL345559	OL191374
	CMK25944_1		OL191244	OL191132	OL191493	OL345560	OL191375
	CMK25944_2		OL191245	OL191133	OL191494	OL345561	OL191376
Schistura tirapensis	3703	Ornamental fish trade	OL191187	OL191057	OL191436	OL345505	OL191301
	3704		OL191188	OL191058	OL191437	OL345506	OL191302

Schistura vinciguerrae	5557	Myanmar, Rakhine prov., Irrawaddy basin, small mountain stream	OL191205	OL191081	OL191454	OL345521	OL191325
	6564<br>6736	Myanmar, Magway division, Irrawaddy basin, Nam Man	OL191211<br>OL191216	OL191087<br>OL191092	OL191460<br>OL191465	OL345527<br>OL345532	OL191331<br>OL191336
	6737<br>6738	Myanmar, Magway division, Irrawaddy basin, Mindon Chaung	OL191217<br>OL191218	OL191093<br>OL191094	OL191466<br>OL191467	OL345533<br>OL345534	OL191337<br>OL191338
Schistura sp. 'Tige.'	9432<br>9433	Thailand, Mae Hong Son prov., Salween basin, River Yuam	OL191232<br>OL191233	OL191119<br>OL191120	OL191481<br>OL191482	OL345548<br>OL345549	OL191363<br>OL191364
Schistura aff. paucicincta	6864	Thailand, Tak prov., Mae Klong basin, Khwae Yai	OL191225	OL191101	OL191474	OL345541	OL191345
Schistura sp. 'Goat Chaung'	3006<br>3008	Myanmar, Rakhine prov., Irrawaddy basin, Goat Chaung	OL191174<br>OL191175	OL191044<br>OL191045	OL191424<br>OL191425	OL345493<br>OL345494	OL191289<br>OL191290
Schistura sp. 'Myanmar'	2573<br>2574	Myanmar, no details	OL191169<br>OL191170	OL191039<br>OL191040	OL191419<br>OL191420	OL345488<br>OL345489	OL191284<br>OL191285
**Physoschistura species group**							
Mustura bella	CMK 26052	Laos, Louang Namtha prov., Mekong basin, Nam Ma	OL191242	OL191130	OL191491	OL345558	OL191373
Mustura geisleri	1238<br>1239	Thailand, Chiang Mai prov., Chao Phraya basin, Mae Taeng; Type locality	OL191158<br>OL191159	OL191023<br>OL191024	OL191407<br>OL191408	OL345477<br>OL345478	OL191270<br>OL191271
Mustura pseudobrunneana	1356<br>1357	Thailand, Chiang Rai prov., Mekong basin, Nam Mae Lao	OL191163<br>OL191164	OL191028<br>OL191029	OL191412<br>OL191413	OL345482<br>OL345483	OL191275<br>OL191276
	850	Chao Phraya basin, River Yom	OL191155	OL191019	OL191403	OL345474	OL191266
Petruichthys brevis	6739<br>6503<br>4184<br>4185	Myanmar, Shan state, Inle lake basin	OL191219<br>OL191210<br>KP738571<br>KP738572	OL191095<br>OL191086<br>OL191063<br>OL191064	OL191468<br>OL191459<br>KP738531<br>KP738532	OL345535<br>OL345526<br>KP738491<br>KP738492	OL191339<br>OL191330<br>OL191307<br>OL191308
Physoschistura brunneana	578	Myanmar, Shan state, Inle lake basin	OL191138	OL191002	OL191386	OL345460	OL191249
Physoschistura shuangjiangensis	2999	China, Yunnan prov., Mekong basin, River Xiohei	OL191173	OL191043	OL191423	OL345492	OL191288
Physoschistura cf. rivolicola	6806	Myanmar, Shan state, stream close to Inle Lake	OL191223	OL191099	OL191472	OL345539	OL191343
Physoschistura rivolicola	6670	Myanmar, Shan state, Inle lake basin, stream Thale-U	OL191214	OL191090	OL191463	OL345530	OL191334
Physoschistura sp	2256	Myanmar, Shan state, Inle Lake basin, unnamed spring	OL191165	OL191033	OL191415	OL345484	OL191280
	7545<br>7546	Aquarium trade	KP738600<br>KP738601	OL191111<br>OL191112	KP738560<br>KP738561	KP738520<br>KP738521	OL191355<br>OL191356
Pteronemacheilus luciodorsum	6695	Myanmar, Shan state, Irrawaddy basin, River Myitnge	OL191215	OL191091	OL191464	OL345531	OL191335

	8465<br>8466	Myanmar, Shan state, Irrawaddy basin, Nam Paw; type locality	KP738606<br>KP738607	OL191116<br>OL191117	KP738566<br>KP738567	KP738526<br>KP738527	OL191360<br>OL191361
Pteronemacheilus sp.	5851<br>5852	Myanmar, Shan state, Irrawaddy, River Myitnge	OL191208<br>OL191209	OL191084<br>OL191085	OL191457<br>OL191458	OL345524<br>OL345525	OL191328<br>OL191329
Schistura crabro	CMK 24559	Laos, Bolikhamsai prov., Mekong basin, Nam Xan	OL191231	OL191118	OL191480	OL345547	OL191362
Schistura kloetzliae	CMK 25994_1<br>CMK 25994_2	Laos, Louang Namtha prov., Mekong basin, Nam Youan	OL191240<br>OL191241	OL191128<br>OL191129	OL191489<br>OL191490	OL345556<br>OL345557	OL191371<br>OL191372
Schistura mahnerti	777<br>803<br>822	Thailand, Mae Hong Son prov., Salween basin, Yuam; inclu. type locality	OL191152<br>OL191153<br>OL191154	OL191016<br>OL191017<br>OL191018	OL191400<br>OL191401<br>OL191402	OL345471<br>OL345472<br>OL345473	OL191263<br>OL191264<br>OL191265
	978	Myanmar, Kayan prov., River Ataran	OL191156	OL191020	OL191404	OL345475	OL191267
	4451	Thailand, Tak prov., Mae Klong basin, River Khwae Yai	OL191195	OL191068	OL191444	OL345513	OL191312
	4840<br>11034<br>11069	Thailand, Tak prov., Salween basin, River Moei	OL191198<br>-<br>OL191247	OL191072<br>-<br>OL191135	OL191447<br>OL191496<br>OL191498	OL345516<br>OL345563<br>OL345565	OL191316<br>OL191378<br>OL191380
	6961	Thailand, Surat Thani prov., River Takua Pa	OL191226	OL191105	OL191475	OL345542	OL191349
	CMK 24942<br>CMK 24850	Myanmar, Tanintharyi prov., River Tenasserim	OL191236<br>OL191237	OL191124<br>OL191125	OL191485<br>OL191486	OL345552<br>OL345553	OL191367<br>OL191368
Schistura pridii	7548<br>7549	Ornamental fish trade	KP738602<br>KP738603	OL191113<br>OL191114	KP738562<br>KP738563	KP738522<br>KP738523	OL191357<br>OL191358
**Part C: Outgroup**							
Schistura savona	7532	Ornamental fish trade	KP738599	OL191110	KP738559	KP738519	OL191354
Barbatula barbatula	8394	Czech Republic, Stredocesky prov., River Elbe	KP738605	OL191115	KP738565	KP738525	OL191359
Lefua costata	6942	Ornamental fish trade	KP738591	OL191104	KP738551	KP738511	OL191348
Nemacheilus binotatus	6926	Ornamental fish trade	KP738586	OL191102	KP738546	KP738506	OL191346
Nemachilichthys ruppelli	4341	Ornamental fish trade	KP738573	OL191067	KP738533	KP738493	OL191311
Paracanthocobitis pictilis	6940	Ornamental fish trade	KP738589	OL191103	KP738549	KP738509	OL191347
Paracanthocobitis zonalternans	5331	Ornamental fish trade	KP738582	OL191080	KP738542	KP738502	OL191324
Schistura aurantiaca	9580	Thailand, Tak prov., Mae Klong basin, Khwae Yai	MK887018	OL191121	OL191503	MK886926	OL191385
Schistura balteata	2554	Ornamental fish trade	MK886971	OL191038	OL191502	MK886880	OL191384

Schistura bolavensis	4618	Laos, Bolaven plateau, no details	KP738575	OL191071	KP738535	KP738495	OL191315
Schistura fasciolata	5302	China - Aquarium trade	KP738581	OL191079	KP738541	KP738501	OL191323
Schistura robertsi	2424	Thailand, Phang Nga prov., River Tham Nang	MK886959	OL191034	OL191501	MK886869	OL191383
Schistura udomritthiruji	1129	Thailand, Phang Nga prov., River Takua Pa	MK886954	OL191021	OL191405	MK886948	OL191268
	2546	Thailand, Ranong prov., Kapoe basin, Thonglang	OL191168	OL191037	OL191418	OL345487	OL191283
	9886 (CMK 24873)	Myanmar, Tanintharyi prov., River Tenasserim	OL191234	OL191122	OL191483	OL345550	OL191365
	9894 (CMK 24849)		OL191235	OL191123	OL191484	OL345551	OL191366
Triplophysa grahami	1663	China, Yunnan, Yangtse basin, River Jinsha	MK608125	OL191030	OL191414	MT536722	OL191277
Triplophysa siluroides	1797	Ornamental fish trade	MT536720	OL191031	EF063156	MT536723	OL191278
Triplophysa strauchi	11496	Kyrgyzstan, Naryn prov., Syrdarja basin, River Naryn	MT536721	OL191137	OL191500	MT536724	OL191382
Cobitis taenia	1860	Germany, Lower Saxonia, Weser basin, River Hunte	EF508508	OL191032	EF056334	MK608315	OL191279

Table S2. Genetic difference (in percent) between lineages in *Schistura poculi* group. Above diagonal mitochondrial genes (Cyt *b* and CO1) and bellow diagonal nuclear genes (RAG 1, IRBP 2, MYH 6).

	Poculi Mekong	Poculi Mae Khlong	Poculi C Yunnan	Tirapen-sis	Calli-dora	Pauci-cincta	Scatu-rigina	Shanen-sis	Siju-ensis	Thavo-nei	Vincigu-errae	S. sp. Myanmar	S. sp. Goat Chaung	S. aff. pauci-cincta	S. sp. Tiger	Outgroup
Poculi Mekong	-	2.18	2.24	5.82	5.65	4.62	6.68	5.22	5.41	4.44	5.22	5.35	4.48	4.52	4.84	7.72
Poculi Mae Khlong	0.14	-	2.69	6.01	6.04	4.65	6.67	5.44	5.49	4.78	5.51	5.93	5.15	4.70	4.91	7.90
Poculi C Yunnan	0.09	0.14	-	5.77	5.25	4.13	6.07	4.83	5.02	4.30	4.52	4.83	4.07	4.34	4.70	7.54
Tirapensis	0.70	0.75	0.69	-	6.22	6.35	6.03	5.89	5.70	5.94	5.30	6.44	6.06	7.02	6.77	7.85
Callidora	0.24	0.29	0.23	0.46	-	6.02	6.46	5.75	6.08	5.56	4.73	5.95	5.58	6.29	6.18	7.56
Paucicincta	0.36	0.41	0.36	0.73	0.27	-	7.08	6.13	6.05	5.41	5.37	6.42	5.48	3.92	2.81	8.23
Scaturigina	0.95	0.99	0.94	0.78	0.71	0.98	-	6.48	4.94	6.30	5.93	6.86	5.96	7.10	8.07	8.08
Shanensis	0.71	0.76	0.71	0.82	0.63	0.75	1.14	-	5.41	5.36	5.08	5.60	4.96	5.80	6.12	7.87
Sijuensis	0.47	0.52	0.46	0.45	0.37	0.50	0.77	0.74	-	5.56	5.28	5.68	5.01	5.64	6.38	7.39
Thavonei	0.32	0.36	0.31	0.69	0.23	0.35	0.94	0.70	0.46	-	4.78	5.15	4.61	5.40	5.72	8.02
Vinciguerrae	0.35	0.40	0.35	0.57	0.27	0.39	0.82	0.62	0.34	0.34	-	5.14	4.32	5.86	6.08	7.17
S. sp. Myanmar	0.79	0.84	0.71	0.78	0.71	0.83	1.03	0.99	0.63	0.78	0.67	-	4.87	6.25	6.77	8.31
S. sp. Goat Chaung	0.37	0.42	0.37	0.59	0.29	0.25	0.84	0.65	0.36	0.36	0.25	0.69	-	5.62	5.97	7.48
S. aff. paucicincta	0.28	0.29	0.27	0.73	0.27	0.16	0.98	0.74	0.50	0.34	0.38	0.82	0.40	-	4.21	8.29
S. sp. Tiger	0.32	0.36	0.31	0.69	0.23	0.04	0.94	0.70	0.46	0.31	0.34	0.78	0.36	0.11	-	8.43
Outgroup	1.44	1.49	1.43	1.49	1.27	1.46	1.72	1.61	1.42	1.43	1.34	1.64	1.31	1.47	1.42	-
